# Zero contrast technique in doubly committed subarterial ventricular septal defect closure in patient with body weight less than 10 kg: a case report

**DOI:** 10.3389/fcvm.2025.1564232

**Published:** 2025-07-02

**Authors:** Radityo Prakoso, Rina Ariani, Aditya Agita Sembiring, Brian Mendel, Oktavia Lilyasari

**Affiliations:** ^1^ Division of Pediatric Cardiology and Congenital Heart Disease, Department of Cardiology and Vascular Medicine, National Cardiovascular Centre of Harapan Kita, Universitas Indonesia, Jakarta, Indonesia; ^2^ Division of Non-invasive Diagnostic and Cardiovascular Imaging, Department of Cardiology and Vascular Medicine, National Cardiovascular Centre of Harapan Kita, Universitas Indonesia, Jakarta, Indonesia; ^3^ Department of Cardiology and Vascular Medicine, National Cardiovascular Centre of Harapan Kita, Universitas Indonesia, Jakarta, Indonesia

**Keywords:** DCSA VSD, small body weight, TTE, zero-contrast, transthoracic echocardiogram

## Abstract

**Introduction:**

The complex anatomy of DCSA VSD, its proximity to valvular and conduction tissues, and concerns about radiation and contrast make transcatheter closure particularly challenging in pediatric patients. While zero-fluoroscopy closure was not achievable in this case, we successfully performed zero-contrast transcatheter closure in a baby weighing less than 10 kg. This report highlights the feasibility and early outcomes of this approach at our institution.

**Case illustration:**

An 18-month-old, 9 kg boy with a history of feeding difficulties, failure to thrive, and breathlessness since 14 days of age underwent transcatheter closure of a doubly committed subarterial (DCSA) ventricular septal defect (VSD). Transthoracic echocardiography revealed a 4–5 mm left-to-right shunting VSD with preserved ventricular function. Initial attempts to cross the VSD under zero-fluoroscopy guidance using various catheters were unsuccessful, necessitating fluoroscopic assistance without contrast. A Konar-MF VSD occluder (7/5 mm) was successfully deployed retrogradely, achieving complete defect closure with no residual shunt or valve dysfunction. Post-procedure, the patient remained asymptomatic, with excellent device positioning and no complications noted at follow-up.

**Conclusion:**

This case demonstrates the feasibility of zero-contrast percutaneous DCSA-VSD closure in selected patients weighing less than 10 kg. Further studies are needed to validate its safety and long-term outcomes.

## 1 Introduction

Ventricular septal defect (VSD) is the most common congenital cardiac malformation, representing approximately 30% of congenital heart disease (1). The highly heterogeneous anatomical morphology of VSD, coupled with its close proximity to the valvular system and conduction tissues, makes transcatheter closure a technically challenging procedure with potential for significant complications (2, 3). In pediatric patients, these procedures are particularly concerning due to their increased vulnerability to the adverse effects of radiation and contrast agents (4–8).

Wang et al. (9) demonstrated that transcatheter closure of perimembranous VSD (pmVSD) under transthoracic echocardiography (TTE) guidance alone is both safe and effective, yielding outcomes comparable to those achieved with fluoroscopic guidance in patients weighing more than 10 kg and with VSD sizes less than 8 mm. However, there are no reported cases of TTE-guided only closure for doubly committed subarterial (DCSA) VSD in patients weighing less than 10 kg.

In this report, we aim to highlight that while complete defect closure using a zero-fluoroscopy technique was not feasible in this case, successful closure was achieved using a zero-contrast technique. We present the efficacy and early outcomes of transcatheter closure of DCSA-VSD in small baby performed at our institution.

## 2 Case illustration

**Figure 1:**
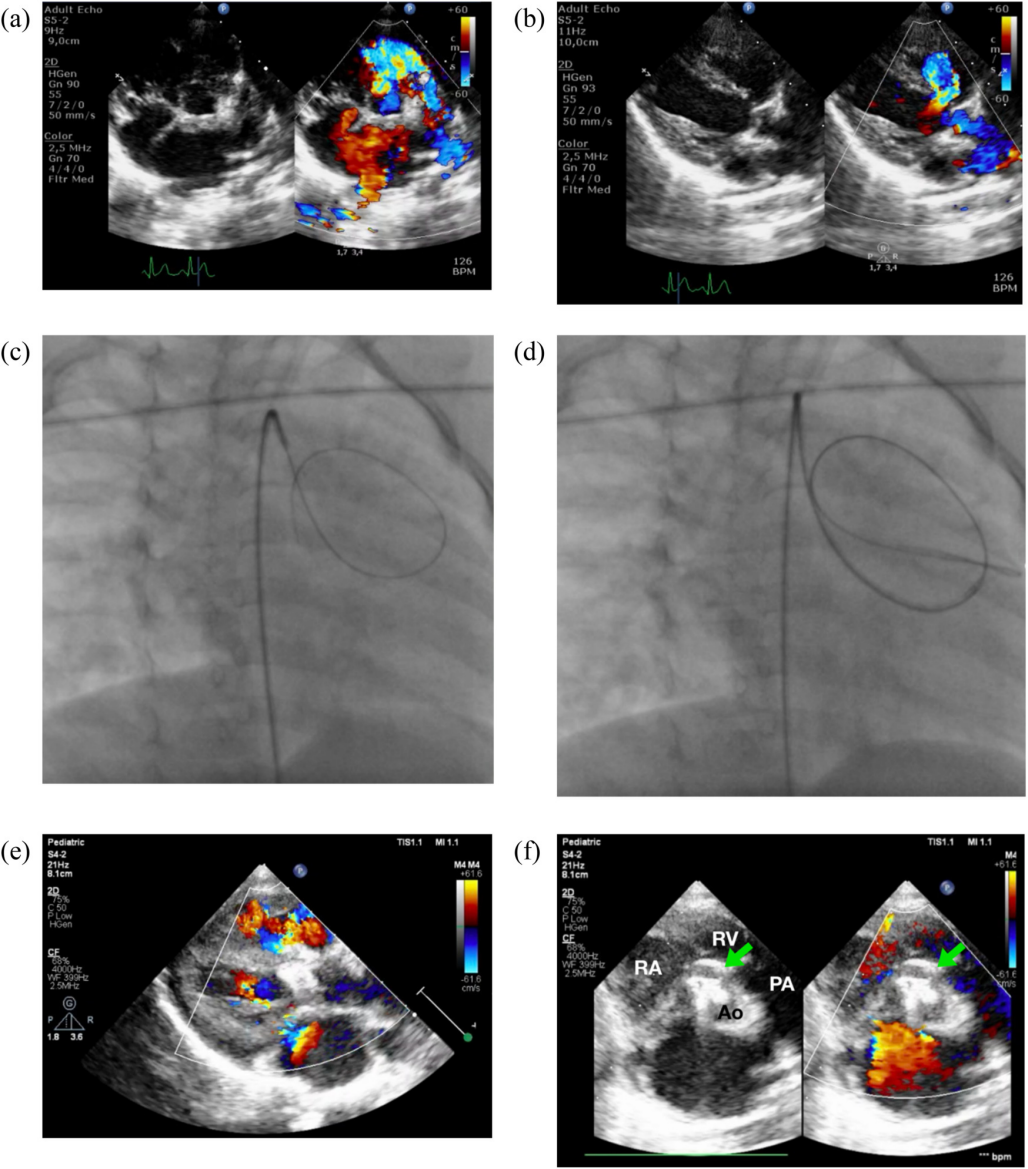
Zero-contrast closure of a doubly-committed subarterial VSD. Transthoracic echocardiography revealed a DCSA VSD in both the parasternal short-axis/PSAX **(a)** and long-axis/PLAX **(b)** views. Simultaneous fluoroscopy with the view of RAO 30/CRAN 0 **(c,d)** and transthoracic echocardiography was employed to cross the VSD by using 3.5/5F JR guiding catheter and 0.035″ soft hydrophilic exchange wire. Following device deployment, imaging confirmed proper device placement without evidence of aortic or tricuspid regurgitation **(e)**. **(f)** PSAX view at aortic level confirmed device edge is touching the pulmonary valve, but no aortic regurgitation was observed. RA, right atrium; RV, right ventricle; PA, pulmonary artery; Ao, aorta.

An 18-month-old boy weighing 9 kg was scheduled for ventricular septal defect (VSD) closure using a device. The patient had a history of feeding difficulties, failure to thrive, and breathlessness, which had been present since the age of 14 days. Initially, these symptoms were attributed to respiratory issues. His vital signs included a blood pressure of 83/40 mmHg, heart rate of 116 bpm, and oxygen saturation of 100%. On physical examination, a palpable thrill was noted at the apex, along with a grade III/VI holosystolic murmur radiating to the axilla. Transthoracic echocardiography revealed a doubly committed subarterial (DCSA) VSD with a left-to-right shunt, measuring 4–5 mm in diameter (Figures 1a,b), and a trans-VSD gradient of 92 mmHg. Left ventricular function was preserved with an ejection fraction (EF) of 65% (Teichholz), and right ventricular function was good with a tricuspid annular plane systolic excursion (TAPSE) of 2.2 cm. The planned intervention was to close the defect using a Konar-MF VSD occluder (size 7/5 mm) via a retrograde transarterial approach under zero-fluoroscopy guidance.

The patient was sedated, and antiseptic preparation was performed on the left and right inguinal regions. A puncture was made in the right femoral artery, and a 5F sheath was inserted. Following this, 900 IU of intra-arterial heparin was administered, and a 3.5/5F JR guiding catheter was advanced through the right femoral artery, descending aorta, aortic arch, and ascending aorta into the left ventricle. Cefuroxime 450 mg was administered intravenously for prophylaxis. An attempt was made to cross the ventricular septal defect (VSD) from the left ventricle (LV) to the right ventricle (RV) using the 3.5/5F JR guiding catheter assisted by a 0.035″ soft hydrophilic exchange wire, but this was unsuccessful. The catheter was then switched to a 5F left internal mammary (LIMA) catheter and a 3.5/4F JR diagnostic catheter, also guided by a soft hydrophilic wire, but the attempts remained unsuccessful. The operator decided to proceed with fluoroscopic guidance, still without the use of contrast. A 3.5/5F JR guiding catheter was reintroduced via the right femoral artery, advanced through the descending aorta, aortic arch, and ascending aorta. Guided by the 0.035″ soft hydrophilic exchange wire, the catheter successfully crossed the VSD from the LV to the RV (Figures 1c,d), with the wire anchored in the pulmonary artery, still with zero contrast technique. A Konar-MF VSD occluder (size 7/5 mm) was deployed through the 3.5/5F JR guiding catheter. The high-pressure disc was positioned in the RV and retracted to seal the VSD, while the low-pressure disc was deployed within the LV to complete the occlusion.

Post-procedural transthoracic echocardiography (TTE) revealed a preserved left ventricular ejection fraction (EF) of 69% (Teichholz) and good right ventricular (RV) contraction. The device was securely positioned, with no residual VSD detected through the device (Figures 1e,f). The cardiac valves were intact, with no evidence of tricuspid regurgitation or aortic regurgitation. A tug test confirmed the stability of the device. The total fluoroscopy time was 3.1 min, with a dose area product (DAP) of 40 mGy. At the most recent follow-up, the patient was asymptomatic, with no reported complications.

## 3 Discussion

### 3.1 Feasibility of doubly committed subarterial ventricular septal defect closure

Closure of ventricular septal defects (VSDs) is commonly performed for perimembranous and muscular types (1, 3, 5); however, the closure of doubly committed subarterial (DCSA) VSD remains controversial due to its proximity to the cardiac valves. Kuswiyanto et al. (2) reported a procedural success rate of 92.5% for transcatheter closure of DCSA-VSD. In their study, the failure rate was 7.5%, primarily due to device dislodgement, moderate residual shunts, and the development of moderate aortic regurgitation (AR). Similar complications, including device dislodgement and new-onset AR, have been reported in other studies as reasons for procedural failure. Accurately measuring the defect size in DCSA-VSD is particularly challenging, especially when the aortic valve is prolapsing. Undersizing the device may lead to dislodgement, while oversizing risks interfering with the aortic valve, potentially causing regurgitation. However, with advancements in technology and the expertise of experienced operators, these complications can be minimized.

The incidence of aortic valve prolapse (AVP) and aortic regurgitation (AR) in DCSA-VSD ranges from 43% to 73% and 24% to 65%, respectively. Both conditions tend to progress over time during follow-up. The presence of pre-existing AVP or AR, even in mild cases, is associated with an increased risk of procedural failure or worsening regurgitation, making these conditions potential exclusion criteria for transcatheter closure. Early intervention to close the defect, prior to the development of aortic valve complications, is therefore recommended (2).

One of the most significant concerns during transcatheter closure of DCSA-VSD is cardiac conduction block. Atrioventricular block (AVB) may develop immediately due to the compressive effect of the device discs or later as a result of inflammation and fibrotic tissue formation around the device. The reported incidence of complete AVB (cAVB) varies between 0% and 5.7% (2). Immediate or early-onset cAVB appears to be more frequent in patients with lower body weight and larger VSDs, potentially due to the characteristics of softer occluders with longer waists, which reduce radial and clamping forces. However, in our case, no conduction block or other complications were observed (1–3).

### 3.2 Zero-contrast technique in DCSA-VSD with low body weight

Our case demonstrates that percutaneous closure of DCSA-VSD using a zero-contrast technique is a feasible, safe, and effective approach. Echocardiography plays a critical role in this technique by providing real-time visualization of valve function and hemodynamics. It facilitates the device closure process by accurately assessing the relationship between the occluder and adjacent valves, as well as identifying any residual shunt (4–8). Echocardiography is an excellent alternative imaging modality, offering high-quality anatomical detail to guide DCSA-VSD closure. While low-osmolar and iso-osmolar iodinated contrast media are commonly used in VSD closure procedures, their use is associated with adverse reactions ranging from mild skin rash and itching to life-threatening anaphylaxis. Additionally, contrast-induced acute kidney injury (CI-AKI) is a significant complication, with an incidence ranging from 3.3% to 14.5% (10). Reducing contrast volume is one of the most effective modifiable strategies to lower the risk of CI-AKI, emphasizing the value of alternative imaging techniques like echocardiography.

The primary challenge in crossing during zero-fluoroscopy VSD closure arises when the VSD angle is less than or equal to 90° relative to the interventricular septum (IVS). Due to the limited visualization in such cases, we decided to convert to a zero-contrast technique. This approach can be performed accurately without risk to the valves, as they are clearly visualized using transthoracic echocardiography. Overall, there is no significant difference in applying this technique between pediatric and adult patients. In our approach, we initially used the reverse technique to minimize the risk of interference with the right coronary cusp by the high-pressure disk (11). However, we later found that device placement is relatively safe for subaortic and doubly committed subarterial (DCSA) VSDs. Consequently, the reverse technique is not strictly necessary in these cases.

Device closure of DCSA-VSD was successfully performed under transthoracic echocardiography (TTE) guidance using a zero-contrast technique without employing an arteriovenous circuit, thereby mitigating the risk of tricuspid valve damage. Furthermore, echocardiography offers the advantage of reducing the likelihood of tricuspid regurgitation by providing real-time visualization of the positioning and deployment of the right-sided disc, ensuring optimal coverage of the right ventricular defect while maintaining a safe relationship with the tricuspid valve and chordae tendineae (4–9). Essentially the location and size decide the size of the device needed. Currently available Konar-MF VSD occluder, provides versatility in its use and their softness and design provide more safety from damage to adjacent structures especially the conduction system (11). Closure at an early age decreases the chance for aortic regurgitation, but then the size of the baby could be a factor limiting the size of the sheath to deliver the device, where in a mismatch could result in compromising the limb blood flow due to oversized vascular access. All these five factors, size of the defect, nearness to the adjacent structures, device related factors, age of the baby, size of the sheath needed for vascular access are discussed in the article. The good transthoracic imaging that can be achieved in the infant pediatric age group, by trained sonographers and state of the art equipment will go a long way in limiting the fluoroscopic imaging time, and the amount of angiographic contrast used.

## 4 Conclusion

Our case showed that percutaneous DCSA-VSD closure can be successfully performed under zero contrast guidance alone in selected patients with weight less than 10 kg. Larger, prospective controlled studies are necessary to further evaluate the feasibility and long-term results of this method.

## Data Availability

The original contributions presented in the study are included in the article/Supplementary Material, further inquiries can be directed to the corresponding author.
